# Correlates of the Lumbar–Femoral T-Score Difference in Older Adults: Sex Outweighs the Triglyceride–Glucose Index

**DOI:** 10.3390/jcm15207773

**Published:** 2026-10-09

**Authors:** Rıdvan Erten, Kübra Erdoğan, Mehmet Poyrazer, Özgür Çakır, Rana Tuna Doğrul, Hande Selvi Öztorun, Kamile Sılay

**Affiliations:** 1 Division of Geriatrics, Department of Internal Medicine, Faculty of Medicine, Kocaeli University, 41380 Kocaeli, Türkiye; 2 Geriatrics Clinic, Ankara Bilkent City Hospital, 06800 Ankara, Türkiye; kubraacmn@hotmail.com (K.E.); rana_tuna@hotmail.com (R.T.D.); 3 Division of Endocrinology and Metabolism, University of Health Sciences Ankara Training and Research Hospital, 06230 Ankara, Türkiye; dr.mehmetpoyrazer@gmail.com; 4 Department of Radiology, Faculty of Medicine, Kocaeli University, 41380 Kocaeli, Türkiye; 5 Division of Geriatrics, Department of Internal Medicine, Faculty of Medicine, Ankara Yıldırım Beyazıt University, 06800 Ankara, Türkiye; drhandeslv@hotmail.com (H.S.Ö.); kamilesilay@hotmail.com (K.S.)

**Keywords:** osteoporosis, T-score discordance, bone mineral density, sex characteristics, aged, triglyceride–glucose index

## Abstract

**Background/Objectives:** We examined correlates of lumbar–femoral T-score differences (ΔT), retaining the triglyceride–glucose (TyG) index as the prespecified primary exposure. **Methods:** This retrospective cross-sectional analysis of routine records included 528 geriatric outpatients assessed in Ankara, Türkiye, in 2023; primary regression used 506 complete cases. ΔT was lumbar minus femoral-neck T-score. Device L1–L4 composites were used without vertebra-level exclusions because scan images were unavailable. Firth models and an exploratory post hoc arithmetic decomposition were secondary. **Results:** Male sex was associated with higher ΔT (B = +0.984; *p* < 0.001), uniquely explaining 9.87% of variance versus 0.88% for TyG. TyG estimates (95% CI) were +0.243 (+0.033 to +0.453; *p* = 0.023) in the primary analysis, +0.214 (−0.010 to +0.438; *p* = 0.061) retaining two records excluded by plausibility limits, and +0.070 (−0.115 to +0.255; *p* = 0.458) excluding influential observations. The male lumbar-worse OR was 0.12 (95% CI 0.03–0.31), based on 3/165 male events. Among patients with femoral-neck osteoporosis, lumbar T-scores exceeded −2.5 in 22/30 men versus 43/89 women (post hoc, unadjusted). **Conclusions:** Sex explained more unique ΔT variance than TyG. The TyG association was small and sensitive to analytic decisions. Unmeasured degeneration and selection bias limit interpretation.

## 1. Introduction

Osteoporosis contributes to disability, morbidity and healthcare costs in ageing populations, and the burden of fragility fractures is increasing [1,2]. Dual-energy X-ray absorptiometry (DXA) of the lumbar spine and proximal femur is a reference method for bone mineral density (BMD) assessment. The T-score expresses the number of standard deviations (SDs) by which a patient’s areal BMD differs from a young-adult reference mean; a T-score ≤ −2.5 defines densitometric osteoporosis [3]. The International Society for Clinical Densitometry (ISCD) recommends assessment of both spine and hip, with classification based on the lowest valid T-score [4].

Disagreement frequently occurs between the two sites. Categorical T-score discordance, in which the lumbar spine and hip fall into different diagnostic categories, is prevalent [5]. Spinal degenerative changes can elevate measured spinal BMD [6,7]. Male sex and diabetes have been associated with spine–femur discordance in an older cohort, with lumbar degeneration, diffuse idiopathic skeletal hyperostosis and aortic calcification proposed as explanations [8]. Men also have higher BMD at both sites, with a larger difference at the lumbar spine [9]. Categorical spine–hip discordance has been reported in men aged ≥50 years in Türkiye [10]. Less is known about the relative contributions of clinical and metabolic correlates to the continuous lumbar–femoral T-score difference (ΔT) in geriatric practice. Continuous spine–hip T-score differences have been studied for fracture-risk prediction [11], but their interpretation also depends on the metric: under a common female reference, site-specific BMD differences are divided by different young-adult reference standard deviations. This arithmetic may affect the magnitude of the sex difference in ΔT without identifying its biological or measurement-related origin.

Increasingly, metabolic factors are being recognised as potentially modifiable correlates of bone status. The triglyceride–glucose (TyG) index, a straightforward proxy for insulin resistance derived from fasting triglyceride and glucose levels [12], has been associated with bone health. Nevertheless, the evidence is inconsistent, as certain population studies report a positive association with total BMD, while others indicate an inverse relationship [13,14,15]. A recent systematic review reported heterogeneous associations, including null findings in several populations aged ≥50 years [13]. Whether insulin resistance, as indexed by the TyG index, contributes to the lumbar–femoral T-score difference in older adults remains unknown and was the prespecified hypothesis of the present study.

These questions are particularly relevant to men. Osteoporosis in men is often not diagnosed or treated adequately, despite nearly 40% of osteoporotic fractures occurring in men and post-fracture mortality being greater than in women [16]. Since 2013, the ISCD has proposed utilising a uniform reference database of white young-adult females for T-score evaluations in males, as a given BMD value measured by DXA carries approximately the same fracture risk for both sexes [17]. The interaction of this guideline-endorsed practice with site-specific measurements of the spine and femur, and whether its diagnostic consequences differ by sex, have not been adequately characterised.

We examined correlates of the continuous lumbar–femoral T-score difference (ΔT = lumbar spine T-score minus femoral-neck T-score) in a single-centre geriatric outpatient cohort, with particular attention to sex differences and their expression under a common reference scale. TyG remained the prespecified primary exposure, and its coefficient in the fully adjusted model remained the single prespecified primary test. The presentation gives greater emphasis to the more consistent sex association without changing that analysis hierarchy. Secondary analyses described categorical discordance; the reference-scaling decomposition was post hoc and exploratory.

## 2. Materials and Methods

### 2.1. Study Design and Setting

This retrospective, single-centre, cross-sectional study used routinely collected records from patients assessed at the geriatric outpatient clinic of Ankara Bilkent City Hospital between January and December 2023. Reporting is guided by STROBE [18] and its RECORD extension [19]; the accompanying checklist (Supplementary File S1) identifies the relevant sections and unavailable documentation. The Clinical Research Ethics Committee approved the retrospective review (TABED 2-26-2331, 13 May 2026), and informed consent was waived because the analysis used de-identified clinical records.

### 2.2. Participants and Eligibility

The screened population comprised 961 geriatric outpatient records. Patients aged ≥ 65 years with paired lumbar and femoral-neck T-scores and the glucose and triglyceride measurements needed to calculate TyG were considered for inclusion. Screening exclusions addressed age < 65 years or missing age, missing core measurements, recorded estimated glomerular filtration rate (eGFR) < 30 mL/min/1.73 m^2^ (CKD-EPI equation [20]), active malignancy and DXA values outside the adopted plausibility limits. Missing eGFR or active-malignancy status did not trigger exclusion. Among the 528 retained participants, eGFR was missing in 5 and active-malignancy status in 38; eligibility on those criteria could therefore not be confirmed from the extract for these records. The analytic cohort comprised 528 patients; complete diabetes and hypertension information further defined the fixed primary regression sample of 506. The supplied screening flags were retained and cross-checked against available fields.

### 2.3. Densitometry and Outcome Definitions

DXA was conducted on a GE Lunar Prodigy Advance densitometer (build PA+503020). The reported device configuration used a common young-adult white-female reference in both sexes (USA NHANES/Lunar database, version 113), consistent with unisex-reference recommendations [4,17]; Z-scores used the Turkish database (version 111). Site reference parameters were back-calculated by regressing BMD on device T-score in patients with paired raw BMD: approximately μ = 1.192 g/cm^2^ and σ = 0.124 g/cm^2^ at the anteroposterior (AP) spine, and μ = 1.037 g/cm^2^ and σ = 0.138 g/cm^2^ at the femoral neck. Unrounded fitted standard deviations were used in the arithmetic reconstruction. The proximal femur was scanned unilaterally, and the femoral-neck region of interest was used. Right and left scans contributed to the pooled analysis, but scan laterality was not retained in the research dataset; side-specific comparisons were therefore unavailable. The lumbar T-score was the device-computed L1–L4 composite. Scan images were unavailable for vertebra-level re-adjudication, and structurally abnormal vertebrae were not excluded.

The primary outcome was continuous ΔT = lumbar spine T-score − femoral-neck T-score; positive values indicate a relatively higher lumbar T-score. Secondary categorical outcomes used osteoporosis T ≤ −2.5 and normal T ≥ −1.0 [3]. Osteoporosis-threshold discordance meant osteoporosis at exactly one of the two sites; femur-worse meant femoral-neck osteoporosis with lumbar T > −2.5; lumbar-worse meant lumbar osteoporosis with femoral-neck T > −2.5. Strict Woodson discordance meant a normal score at one site and osteoporosis at the other [5]. These definitions were re-derived from the recorded T-scores and checked against the stored flags.

### 2.4. Primary Exposure and Covariates

TyG was calculated as ln[fasting triglyceride (mg/dL) × fasting glucose (mg/dL)/2], using the formulation reported by Zhan et al. [15]. The primary covariates were age, sex, diabetes mellitus and hypertension. Diabetes and hypertension were represented by the binary diagnoses in the research extract, with missing entries left missing. The extract did not retain the original hospital coding algorithms, diagnostic look-back periods or validation metadata. Table S7 documents the available research fields and derived-variable definitions; unavailable source coding information is identified explicitly in the RECORD checklist.

### 2.5. Laboratory Data

Glucose and triglyceride concentrations were expressed in mg/dL for calculation of TyG. Serum 25-hydroxyvitamin D was descriptive only and is reported in nmol/L from the harmonised source field. Descriptive summaries used observed non-missing values, with a denominator shown for every row; missing values were not imputed for Table 1.

### 2.6. Data Quality Control

The data-cleaning rule excluded records with femoral-neck BMD outside 0.30–1.50 g/cm^2^ or an absolute T-score > 6 at any measured site. These plausibility limits were not prespecified in the study protocol. Two records were flagged, with femoral-neck BMD values of 2.305 and 0.080 g/cm^2^. For these two patients, DXA had been performed at an external centre and only the reported values had been transcribed into the clinical notes; because the scan images were unavailable, the underlying acquisition, analysis or recording error could not be adjudicated. For all other participants, DXA was performed in-house and recorded values were verified against the original scan reports. The primary analysis excluded these records, and a sensitivity analysis retained them. This rule was distinct from the regression influence analysis and did not establish that all influential observations were erroneous. For rank-based analyses, ΔT was recomputed from the source T-scores and standardised to two decimal places to prevent floating-point differences from splitting mathematically equal values. Two recorded T-scores had two decimal places; they were preserved rather than rounded to one decimal.

During revision, a post hoc audit identified unit discrepancies, scale-coding problems and inconsistent fields that required source review. The accompanying cell-level correction log records amendments within the analytic cohort to two glucose values (24 to 124 and 26 to 126 mg/dL), one femoral-neck T-score (re-read from the Neck row of the DXA report), one femoral-neck BMD value, one body weight (22.2 to 52 kg), two HbA1c values confirmed as IFCC mmol/mol and converted to NGSP % (40 to 5.8 and 43 to 6.1), together with conversion of 54 albumin values from g/dL to g/L. The log distinguishes source-reviewed corrections from unverified values set to missing and deterministic re-derivations. The unverified analytic-cohort values comprised two Clinical Frailty Scale entries coded 0 and the total-hip T-score of one participant, whose stored value disagreed with the recorded total-hip BMD and with the verified femoral-neck T-score. The log also documents corrections among excluded records. TyG, ΔT and discordance flags were re-derived after these changes. These procedures were not prespecified; the analytic cohort (*N* = 528) and primary complete-case sample (*n* = 506) were unchanged. The separate validation script distinguishes structural inconsistencies from review flags and documented exclusions; a review flag alone does not establish that a value is erroneous.

### 2.7. Statistical Analysis

Continuous variables in Table 1 were summarised as median [interquartile range (IQR)] and compared with two-sided Mann–Whitney U tests; mean (SD) additionally describes ΔT in the text. Binary variables were summarised as *n*/*N* (%) and compared with Pearson chi-square tests with Yates correction, or Fisher exact tests where an expected cell was <5. Mann–Whitney analyses used asymptotic inference with tie and continuity corrections. Patients with available raw BMD were compared with the remainder on observed baseline variables and ΔT; a non-significant comparison was not treated as evidence of equivalence or representativeness. Missingness in screened records was described by variable and joint pattern and compared by age and sex. Additional comparisons among excluded records with paired T-scores were exploratory and were not interpreted as bounds on selection bias.

All hierarchical regression blocks used the same complete-case sample (*n* = 506): Model 1 included age and sex; Model 2 added diabetes and hypertension; Model 3 added TyG. The TyG block in Model 3, assessed by a nested F-test, remained the single prespecified primary test. Relative contributions were summarised by standardised coefficients and drop-one semi-partial R^2^, representing each predictor’s unique contribution within this model. These values do not sum to model R^2^ when predictors are correlated. HC3 heteroscedasticity-consistent standard errors were also calculated, and variance inflation factors (VIFs) assessed linear multicollinearity.

The sample-size assumptions reported for the study were a small incremental effect of the single primary predictor (Cohen’s f^2^ = 0.02), five predictors, α = 0.05 and 80% power. Recalculation during revision using the non-central F distribution indicated a minimum of 395 participants; the original G*Power (version 3.1.9.7) output was unavailable. Observed f^2^ was calculated from the increment in R^2^ divided by 1 − R^2^ of the full model. This descriptive effect size was not used as a post hoc justification of power.

Diabetes was retained in the primary model because it may be associated with bone through pathways not captured by TyG. However, diabetes may also partly represent the glucose component of TyG, and its position relative to the exposure and outcome cannot be resolved in this cross-sectional analysis. Low VIFs address multicollinearity rather than the possibility of overadjustment, collider bias or residual confounding. The diabetes-omitted model was therefore reported as a sensitivity analysis on the same 506 participants.

Sensitivity analyses examined HC3 inference; retention of the two records excluded by plausibility limits; exclusion of observations with Cook’s distance > 4/*n*; omission of diabetes; addition of body mass index (BMI), height, smoking and alcohol, or clinical frailty; total-hip rather than femoral-neck ΔT; recorded osteoporosis treatment; and availability of raw BMD. Models compared before and after adding a covariate used the same subgroup wherever the change was interpreted. Quadratic and tertile TyG analyses were exploratory. Osteoarthritis adjustment, missingness comparisons and the expanded screened-record analyses were added during revision.

BMI multiple imputation was reanalysed during revision using 50 posterior-sampling imputations of log(BMI), conditional on ΔT, age, sex, diabetes, hypertension and TyG. Following the chained-equations framework [21], the implementation used scikit-learn IterativeImputer with a BayesianRidge estimator, posterior sampling, 10 iterations and seeds 0–49. Imputed values were exponentiated before fitting the BMI-adjusted primary model. Estimates and covariance matrices were pooled using Rubin’s rules [22], with Barnard–Rubin degrees of freedom. The log transformation preserved positive BMI support. This newly documented specification replaces the earlier incompletely documented imputation result. It addresses the 158 missing BMI values within the 506 primary complete cases and does not address missing DXA or selection from the screened population.

Categorical outcomes were analysed by Firth-penalised logistic regression [23], adjusted for age, sex, diabetes, hypertension and TyG in the 506 complete cases. The revision implementation maximised the logistic log likelihood plus one half of the log determinant of the Fisher information matrix. Confidence intervals (CIs) and tests were based on the profile penalised likelihood, retaining the full information penalty during constrained fits. Model-specific event counts accompany the estimates. Penalisation does not remove the information limitation from very few male lumbar-worse events.

The exploratory post hoc arithmetic decomposition used unadjusted sex differences in mean raw BMD from the 130 participants with paired BMD. The predicted sex difference in ΔT was the lumbar male–female BMD difference divided by the lumbar reference SD, minus the corresponding femoral-neck term. The common young-adult means cancel in this contrast. A common-SD anchor illustrated the partition into a between-site BMD-difference component and an SD-scaling component; the percentage split depends on this anchor and is descriptive. A reference-free log(lumbar BMD/femoral-neck BMD) model was adjusted for the same five predictors in participants with complete data. This analysis cannot distinguish biology, body size and degenerative artefact.

The analyses were executed in Python 3.12 using NumPy 2.3.5, pandas 2.2.3, SciPy 1.17.0 and scikit-learn 1.8.0; exact runtime versions and seeds are recorded in the accompanying notebook. The Firth implementation was checked against analytic special cases, an independent optimisation method and profile-likelihood endpoint conditions. Two-sided *p*-values and 95% confidence intervals are reported; *p* < 0.05 was the prespecified significance threshold for the primary test.

## 3. Results

### 3.1. Participant Flow and Cohort Characteristics

Of 961 screened records, 7 were excluded for age (6 aged <65 years and 1 with missing age) and 365 for missing core measurements, leaving 589 for further eligibility assessment. Twenty-one had eGFR < 30 mL/min/1.73 m^2^ and forty had active malignancy; two met both criteria. Two further records were excluded by DXA plausibility limits. The analytic cohort comprised 528 patients: 354 women (67.0%) and 174 men (33.0%). The primary complete-case sample contained 506 patients (diabetes missing in 20 and hypertension in 10, with overlap). Mean ΔT was +0.628 (SD 1.448). Paired raw BMD was available in 130 patients, total-hip T-score in 127, and BMI in 361.

Of the 433 excluded records, 124 had paired lumbar and femoral-neck T-scores. The other 309 lacked paired scores: 291 had both fields missing, 8 lacked only lumbar T-score and 10 lacked only femoral-neck T-score. DXA-field missingness was more frequent in men; no clear sex association was identified for the laboratory or comorbidity fields. Missingness comparisons by age and sex and joint patterns are reported in Table S6.

Adding all 124 excluded records with paired T-scores changed the age-adjusted sex coefficient from +1.014 to +0.976. The pooled age-adjusted model used 651 rather than 652 records because one excluded patient lacked age. This expanded comparison included patients excluded for other clinical or quality criteria and does not estimate or bound selection bias. A comparison restricted to available paired records meeting the clinical and quality criteria is also provided in Table S6.

### 3.2. Baseline Characteristics by Sex

**Figure 1:**
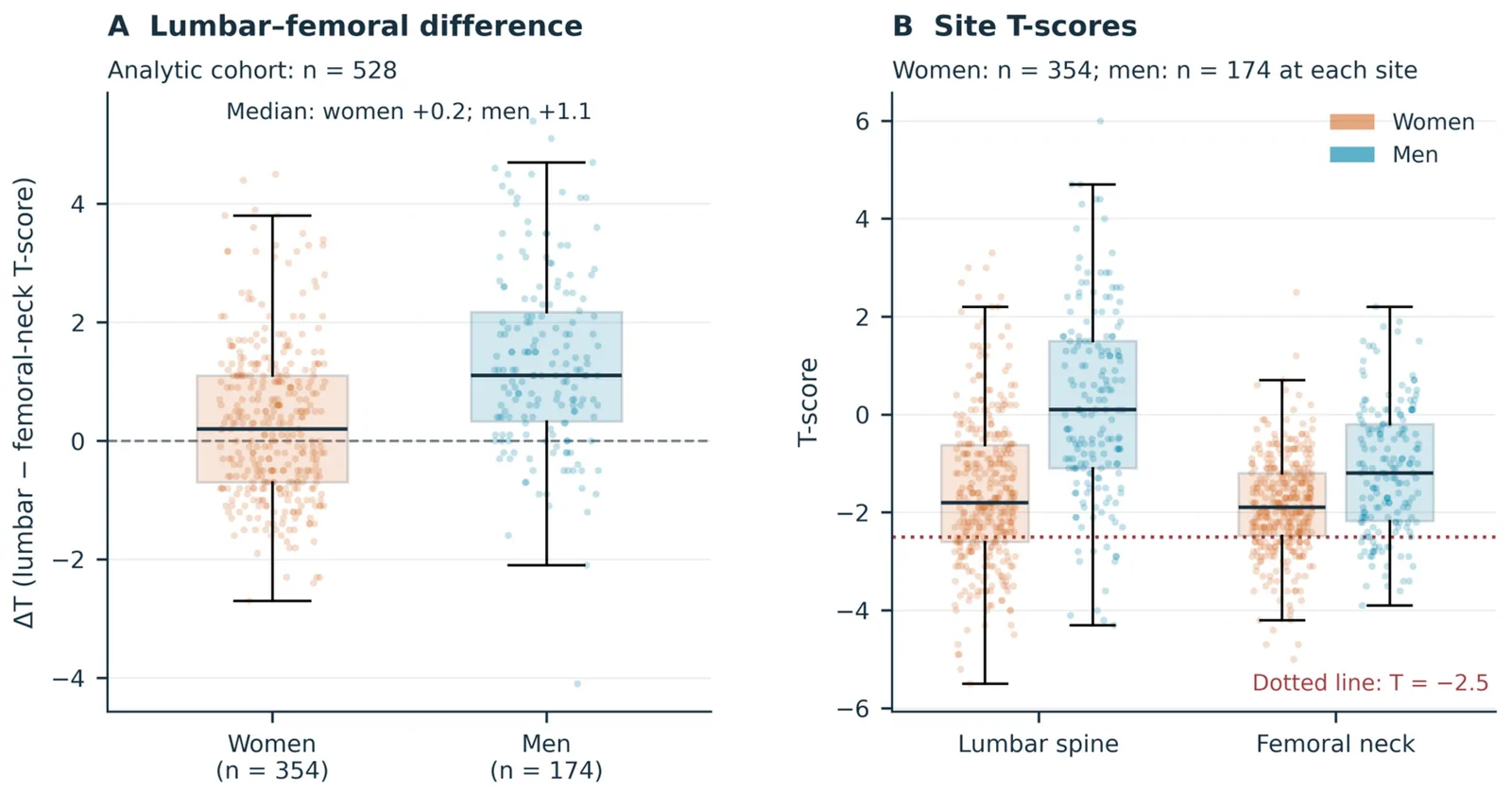
Lumbar–femoral T-score difference and site T-scores by sex in the analytic cohort (*n* = 528; 354 women and 174 men). (**A**) ΔT, defined as lumbar minus femoral-neck T-score; the dashed line marks ΔT = 0. (**B**) Site-specific T-scores; the dotted line marks T = −2.5. In both panels, dots represent individual participants with horizontal jitter only; boxes show the median and interquartile range, and whiskers extend to the most extreme values within 1.5 interquartile ranges.

Men were older (median 78.5 versus 76.0 years), taller and had lower BMI than women. Median lumbar T-score was +0.1 in men versus −1.8 in women, whereas femoral-neck medians were −1.2 versus −1.9. Consequently, median ΔT was +1.1 versus +0.2 (*p* < 0.001). TyG did not show a clear sex difference. Recorded hypertension was more frequent in women (259/350, 74.0%) than men (104/168, 61.9%; *p* = 0.007); hypertension was included in the adjusted models. Descriptive sample sizes vary with item availability (Table 1; Figure 1).

### 3.3. Primary Analysis

Model R^2^ increased from 0.110 for age and sex to 0.138 after adding diabetes and hypertension, and to 0.147 after adding TyG. Male sex had B = +0.984 (95% CI +0.730 to +1.238; *p* < 0.001) and semi-partial R^2^ = 0.0987, corresponding to 9.87% of total ΔT variance. TyG had B = +0.243 (95% CI +0.033 to +0.453; nested F-test *p* = 0.023; HC3 *p* = 0.032) and semi-partial R^2^ = 0.0088, or 0.88% of total variance. The observed incremental f^2^ was approximately 0.010, below the planning value of 0.02. Diabetes was positively associated with ΔT (B = +0.343; *p* = 0.013); the hypertension estimate was imprecise (B = +0.116; *p* = 0.381); recorded hypertension was more frequent in women (74.0% versus 61.9%), and this imbalance is adjusted for rather than removed when that imprecisely estimated coefficient is interpreted. VIFs were 1.06 for age, 1.03 for sex, 1.27 for diabetes, 1.05 for hypertension and 1.22 for TyG; these did not indicate substantial multicollinearity but do not establish the causal appropriateness of adjustment (Table 2).

### 3.4. Robustness

The primary TyG estimate was +0.243 (95% CI +0.033 to +0.453; *p* = 0.023). Retaining the two records excluded by plausibility limits gave B = +0.214 (95% CI −0.010 to +0.438; *p* = 0.061) in 508 participants. Excluding the 33 observations with Cook’s distance > 4/506 gave B = +0.070 (95% CI −0.115 to +0.255; *p* = 0.458) in 473 participants. These findings indicate sensitivity to analytic decisions; crossing a significance threshold alone was not interpreted as disappearance of an association.

Omitting diabetes in the same 506 participants gave B = +0.348 (95% CI +0.154 to +0.542; *p* < 0.001). Restriction to patients without documented active osteoporosis treatment gave B = +0.276 (95% CI +0.064 to +0.488; *p* = 0.011). The BMI-adjusted complete-case result was B = +0.209 (95% CI −0.027 to +0.446; *p* = 0.082; *n* = 348); the revised multiple-imputation analysis gave B = +0.240 (95% CI +0.030 to +0.450; *p* = 0.025) in 506 participants (Table S1).

The smoking/alcohol-adjusted (*n* = 103) and frailty-adjusted (*n* = 108) TyG estimates were imprecise, with intervals including both zero and the primary estimate. The frailty-adjusted sex coefficient was +0.574 (95% CI −0.026 to +1.175; *p* = 0.061). These analyses provide limited additional evidence and do not exclude residual confounding. Height adjustment in the same 358 participants reduced the sex coefficient from +1.086 to +0.711 (95% CI +0.333 to +1.090). Same-subgroup comparisons are provided in Tables S1 and S2, and the sensitivity estimates for both coefficients are summarised in Figure S1.

Recorded osteoarthritis was present in 19/329 women (5.8%) and 6/161 men (3.7%; Fisher exact *p* = 0.389; the Yates-corrected chi-square reported in Table 1 gives *p* = 0.454). Within the same 490 participants, adding osteoarthritis changed the sex coefficient from +0.961 to +0.960. The osteoarthritis coefficient was B = −0.094 (95% CI −0.639 to +0.452; *p* = 0.736). The field did not identify anatomical site or severity and was a limited proxy for lumbar degeneration.

Total-hip T-score was available in 127 participants, of whom 106 had complete model covariates. Within these same 106 participants, the sex coefficient was +0.647 (95% CI +0.043 to +1.251) for lumbar minus femoral-neck T-score and +0.331 (95% CI −0.295 to +0.958) for lumbar minus total-hip T-score. The paired coefficient difference, estimated by regressing total-hip minus femoral-neck T-score on the same covariates, was +0.315 (95% CI +0.040 to +0.591; *p* = 0.025). The point estimate indicates a larger male excess against the femoral neck than against the total hip, but the subset is small and selected, and the comparison does not identify an anatomical or reference-scaling mechanism. Quadratic and tertile TyG estimates are reported in Table S4; neither analysis established a clear nonlinear pattern.

### 3.5. Site Discordance

**Figure 2:**
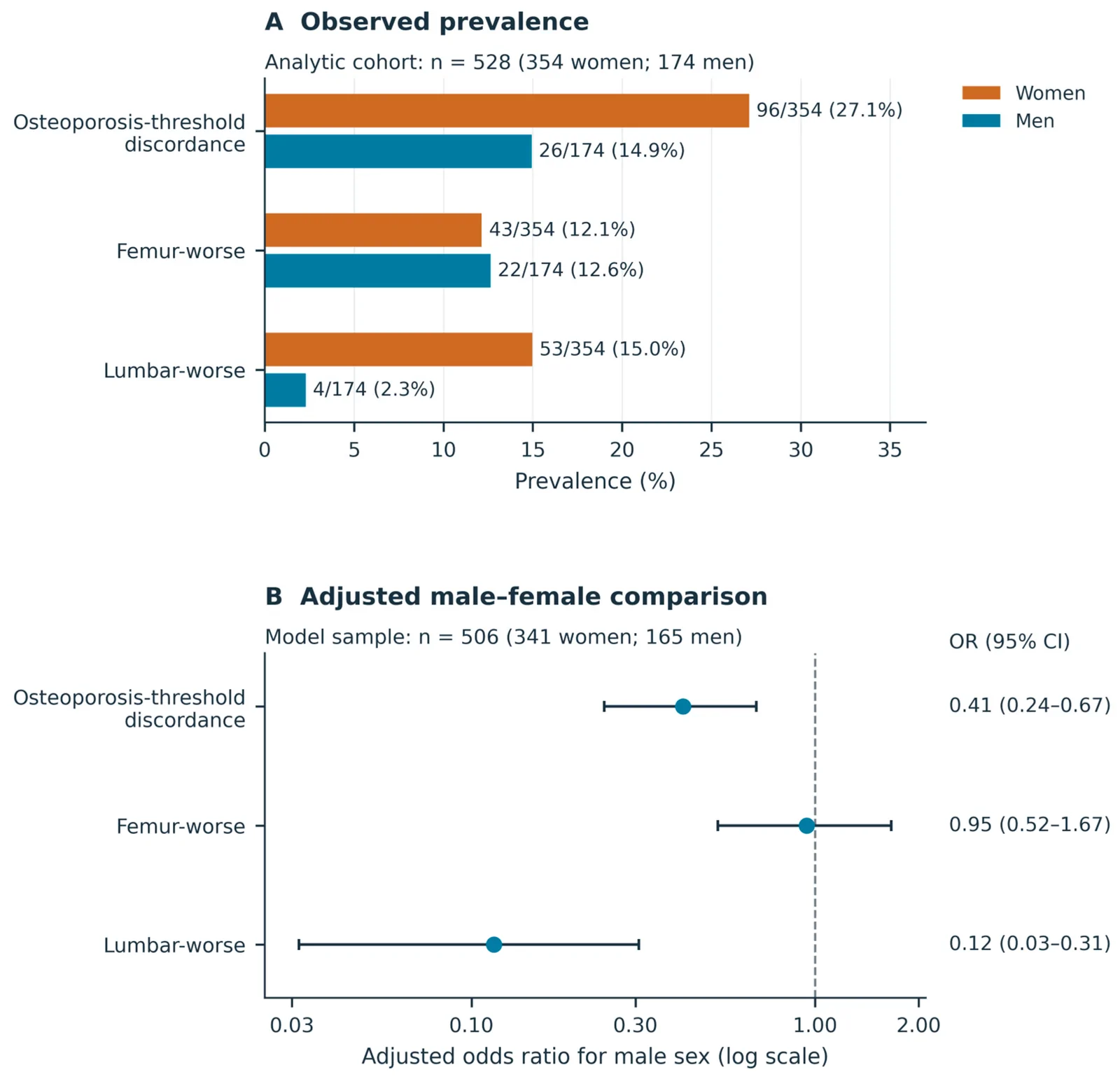
Osteoporosis-threshold discordance by sex. (**A**) Unadjusted prevalence in the analytic cohort (*n* = 528); labels give event counts, denominators and percentages. (**B**) Adjusted odds ratios for male sex from Firth-penalised logistic regression (*n* = 506; 341 women and 165 men), adjusted for age, diabetes, hypertension and TyG. Points and bars indicate odds ratios and 95% profile penalised-likelihood confidence intervals. The dashed line denotes OR = 1. The three outcomes have 115, 60 and 55 model events, respectively. Lumbar-worse discordance occurred in 4/174 men and 53/354 women in the full cohort; the adjusted model included 3/165 male and 52/341 female events. Femur-worse and lumbar-worse denote a T-score ≤ −2.5 at only the indicated site.

Osteoporosis-threshold discordance occurred in 122/528 patients (23.1%). Lumbar-worse discordance occurred in 4/174 men (2.3%) and 53/354 women (15.0%). The adjusted models included 165 men and 341 women, with 3 male and 52 female lumbar-worse events. Male sex was associated with lower odds of osteoporosis-threshold discordance (OR 0.41, 95% CI 0.24–0.67) and lumbar-worse discordance (OR 0.12, 95% CI 0.03–0.31). The femur-worse estimate was OR 0.95 (95% CI 0.52–1.67), providing no clear evidence of a sex difference. In a post hoc, unadjusted comparison among 119 patients with femoral-neck T ≤ −2.5, lumbar T remained >−2.5 in 22/30 men (73.3%) and 43/89 women (48.3%; two-sided Fisher exact *p* = 0.020). This describes threshold classification among patients selected for femoral-neck osteoporosis; it is not a diagnostic-performance or fracture-prediction analysis (Table 3; Figure 2).

### 3.6. Exploratory Arithmetic Decomposition of the Sex Effect (Post Hoc)

The raw-BMD subset contained 130 participants (89 women and 41 men). It differed from the remainder on several baseline characteristics; the ΔT comparison gave *p* = 0.779 and the sex comparison *p* = 0.773 (Table S5), which do not establish equivalence or representativeness. Unadjusted sex differences in BMD and the fitted, unrounded site reference SDs reconstructed a ΔT difference of +0.787, compared with the observed +0.766 in this subset.

**Figure 3:**
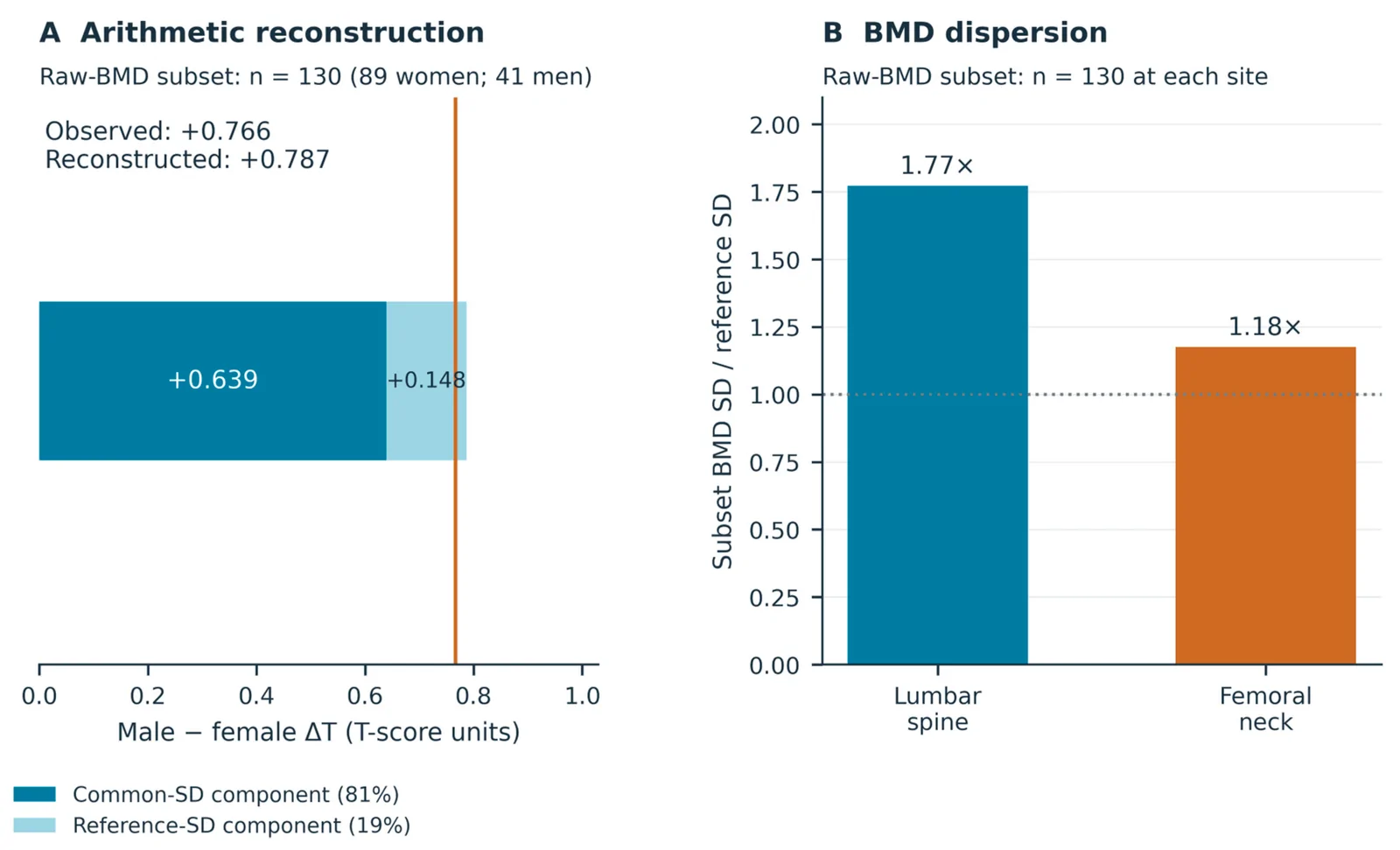
Exploratory post hoc arithmetic reconstruction in participants with raw BMD at both sites (*n* = 130; 89 women and 41 men). (**A**) The unadjusted male–female difference in ΔT was +0.766; the reconstruction from BMD differences and fitted, unrounded reference SDs was +0.787; the vertical orange line marks the observed difference. The common-SD anchor is the arithmetic mean of the two fitted reference SDs. The resulting between-site BMD-difference (common-SD) and SD-scaling (reference-SD) components are approximately 81% and 19%; the sex-stratified bootstrap 95% interval for the between-site BMD-difference share is 51–89% (2000 resamples). These shares depend on the anchor and are not causal contributions. (**B**) Raw-BMD subset SDs divided by the corresponding fitted reference SDs. The dotted line denotes a ratio of 1. The reference parameters were back-calculated by pooled BMD-on-T-score regression; agreement is an arithmetic property of the scale and does not identify the origin of the BMD differences.

Using the mean of the fitted lumbar and femoral-neck reference SDs as the common anchor, approximately 81% of the reconstruction was attributed to the between-site BMD-difference component and 19% to SD scaling. The bootstrap interval for the first share was 51–89% (2000 resamples stratified by sex, with calibration re-estimated in each resample); using either site SD as the anchor changed the point allocation. These are exploratory arithmetic components rather than causal proportions (Figure 3).

The reference-free log(lumbar BMD/femoral-neck BMD) model used 108 participants with complete covariates. Its adjusted male coefficient was B = +0.017 (95% CI −0.056 to +0.091; *p* = 0.637). The wide interval leaves uncertainty about relative BMD differences and cannot distinguish biological variation from body size or degenerative artefact.

## 4. Discussion

Sex was the strongest correlate of continuous ΔT among the variables in the primary model, with approximately one T-score unit higher ΔT in men and a unique contribution of about 10% of outcome variance. TyG contributed less than 1% and its estimate was sensitive to analytic decisions. The title’s comparison is restricted to these within-model contributions; it does not alter TyG’s prespecified primary-test status. The categorical analyses showed fewer lumbar-worse classifications in men, but the adjusted OR of 0.12 (95% CI 0.03–0.31) rested on only 3 male events among 165 men in the model sample. This sparse-event estimate warrants caution despite penalisation.

Under a common reference, sex differences in BMD are expressed on the T-score scale through site-specific standard deviations. The arithmetic reconstruction describes this transformation; it does not explain why lumbar BMD differs more by sex than femoral-neck BMD. Most of the illustrated reconstruction reflected the between-site BMD-difference component, while reference-SD asymmetry contributed a smaller, anchor-dependent component. Similar sex associations have been reported with other outcome and reference definitions [8]. Body size, areal-BMD geometry, degeneration and biological differences remain competing explanations, and close numerical reconstruction is not evidence that these alternatives have been excluded.

The post hoc threshold comparison illustrates why discordant measurements should be considered together: among participants with femoral-neck osteoporosis, a lumbar score above −2.5 was more frequent in men. This selected, unadjusted comparison does not measure the sensitivity of a screening strategy, establish underdiagnosis in the population or quantify fracture risk. No fracture outcome was analysed.

A recorded osteoporosis diagnosis was less frequent in men than women, but this comparison combines differences in underlying prevalence, referral, documentation and treatment history. It cannot by itself quantify underdiagnosis. The broader literature identifies gaps in osteoporosis recognition and treatment in men [16]; the present data add a description of site-specific T-score patterns rather than a population estimate of those gaps.

The findings are compatible with existing recommendations to assess valid spine and hip measurements together [4]. Where spinal degeneration may distort the measurement, appropriate vertebral exclusions and assessment at other valid sites are important [4,6,7]. Clinical assessment also incorporates fragility-fracture history and absolute fracture probability, for which tools such as FRAX are available [24]. This study did not test a new diagnostic strategy or a fracture-risk model.

The TyG finding should be interpreted in the context of inconsistent prior evidence [13,14,15]. Retaining the two records excluded by plausibility limits modestly reduced the estimate, whereas removing influential observations produced a larger reduction. The wide intervals in the smoking/alcohol and frailty subsets were compatible with both the primary estimate and no association; those analyses do not establish that adjustment eliminated the association. Taken together, the results support a small, specification-sensitive association and do not support a causal metabolic explanation for ΔT.

Omitting diabetes increased the TyG coefficient from +0.243 to +0.348 in the same 506 participants. Diabetes may represent skeletal pathways not fully captured by TyG, but it may also overlap with the exposure’s glucose component. Its role as a confounder, intermediate or proxy depends on a causal structure that this cross-sectional study cannot establish. Low VIFs do not resolve that issue. The diabetes-adjusted model remains primary, and the diabetes-omitted estimate is presented as a sensitivity result.

The study extends previous reports of categorical spine–hip discordance [8,10] by examining continuous ΔT and comparing the unique contributions of sex and a prespecified metabolic exposure in a geriatric clinical cohort. The exploratory decomposition clarifies how BMD differences are represented on the common T-score scale. Neither the decomposition nor the relative variance contributions establish biological mechanisms or generalise the estimated magnitudes beyond the measured cohort.

The fixed complete-case sample across hierarchical blocks prevents changes in sample composition from being mistaken for incremental explained variance. Reporting HC3 inference, influence diagnostics, alternative covariate models and explicit data-quality sensitivity analyses helps show which findings depend on modelling choices. The supplied analysis files document the revision calculations and their assumptions.

Several limitations constrain interpretation. The cross-sectional, single-centre design precludes causal inference and limits generalisability to other clinical settings, devices and reference implementations. Although the sex association was consistent across the larger analytic specifications, it remains vulnerable to residual confounding and selection.

Lumbar degeneration could not be assessed radiographically, and the device L1–L4 composite was used without vertebra-level exclusions. Recorded osteoarthritis lacked anatomical site and severity information; adjustment for this variable cannot exclude distortion of lumbar BMD by degenerative change. Scan laterality was also unavailable, so whether side selection differed by sex or was related to ΔT could not be tested. The direction and magnitude of any associated bias are unknown.

Missing measurements excluded 365 screened records at that stage of screening, and 433 records in total did not enter the analytic cohort. Men constituted 41.1% of all excluded records versus 33.0% of included records (Table S3). Paired lumbar and femoral-neck T-scores were absent in 309 records, including 291 with both fields missing and 18 with one site available. Observed missingness was compatible with differences in referral or record availability, but the observed data alone cannot distinguish between missing-at-random (MAR) and missing-not-at-random (MNAR) mechanisms. Adding excluded records with paired T-scores is an exploratory comparison, not an estimate or upper bound of selection bias; the expanded group also includes patients who did not meet other eligibility or quality criteria. For those without paired T-scores, the direction and magnitude of selection bias remain unknown.

The raw-BMD decomposition used 130 patients who differed from the remainder on several baseline characteristics. A non-significant ΔT or sex comparison does not establish representativeness. The arithmetic identity holds for the specified metric, but the estimated component sizes and the anchor-dependent percentage split need not transfer to other patients.

Height adjustment reduced the sex coefficient in the same subgroup, which is compatible with a contribution from body size or the size dependence of areal BMD; it does not identify a causal fraction. The reference-free log-ratio model and the smoking/alcohol and frailty analyses had wide confidence intervals. Residual confounding by body size, smoking, alcohol, frailty and unrecorded treatments remains possible.

The adjusted lumbar-worse analysis contained only three male events, limiting the stability and precision of this contrast. The post hoc threshold comparison was unadjusted, and neither fracture outcomes nor a diagnostic-performance endpoint was analysed. The interval between laboratory sampling and DXA was not reliably available. Glucocorticoid, androgen-deprivation and aromatase-inhibitor exposure were not separately characterised.

The research extract did not preserve complete hospital diagnostic coding, ascertainment-window or validation metadata. Available field definitions and cleaning rules are reported, but this limitation prevents complete reconstruction of the original clinical ascertainment algorithms. Five retained participants had no recorded eGFR and thirty-eight had no recorded active-malignancy status, so their eligibility on those criteria could not be confirmed from this extract. The RECORD checklist identifies these documentation gaps.

## 5. Conclusions

In this geriatric outpatient cohort, male sex was associated with a higher lumbar–femoral T-score difference (ΔT) and accounted for more unique outcome variance than TyG within the primary model. The prespecified TyG association was small and sensitive to analytic decisions. Exploratory reference scaling described the arithmetic expression of BMD differences without resolving their biological or measurement-related origin. Unmeasured lumbar degeneration and selection bias remain important limitations. The descriptive findings are consistent with considering valid spine and hip measurements together, but do not establish diagnostic performance or fracture risk.

## Figures and Tables

**Table 1:** Baseline characteristics of the analytic cohort by sex.

Variable	All (*N* = 528)	Women (*n* = 354)	Men (*n* = 174)	*p*
Age, years	76.0 [72.0, 82.0] *n* = 528	76.0 [71.0, 81.0] *n* = 354	78.5 [73.2, 83.0] *n* = 174	0.002
Height, cm	160.0 [154.5, 165.0] *n* = 371	155.0 [150.0, 160.0] *n* = 243	168.0 [162.0, 172.0] *n* = 128	<0.001
Weight, kg	72.0 [62.0, 80.1] *n* = 371	70.0 [60.0, 80.0] *n* = 246	75.0 [65.7, 85.0] *n* = 125	0.003
BMI, kg/m^2^	27.9 [24.3, 31.5] *n* = 361	28.6 [24.6, 33.2] *n* = 238	26.7 [23.7, 29.8] *n* = 123	<0.001
Glucose, mg/dL	103.0 [90.0, 128.0] *n* = 528	103.5 [90.0, 124.0] *n* = 354	103.0 [90.0, 135.8] *n* = 174	0.786
Triglyceride, mg/dL	123.0 [91.0, 171.2] *n* = 528	123.0 [92.0, 171.7] *n* = 354	127.0 [89.0, 170.8] *n* = 174	0.907
TyG index	8.8 [8.4, 9.2] *n* = 528	8.8 [8.4, 9.2] *n* = 354	8.9 [8.4, 9.3] *n* = 174	0.674
HbA1c, %	6.0 [5.7, 6.9] *n* = 458	6.0 [5.7, 6.8] *n* = 311	6.0 [5.6, 7.0] *n* = 147	0.942
eGFR, mL/min/1.73 m^2^	71.0 [55.1, 84.0] *n* = 523	71.0 [55.6, 85.0] *n* = 349	70.5 [54.3, 83.0] *n* = 174	0.257
Albumin, g/L	43.7 [41.0, 45.0] *n* = 521	44.0 [41.0, 45.1] *n* = 348	43.0 [41.0, 45.0] *n* = 173	0.114
Calcium, mg/dL	9.5 [9.1, 9.8] *n* = 522	9.5 [9.1, 9.9] *n* = 351	9.4 [9.1, 9.8] *n* = 171	0.142
25(OH)D, nmol/L	32.0 [22.0, 53.0] *n* = 465	31.6 [21.0, 53.8] *n* = 314	32.0 [22.0, 50.0] *n* = 151	0.884
TSH, mIU/L	1.6 [1.0, 2.4] *n* = 510	1.7 [1.0, 2.6] *n* = 341	1.4 [0.9, 2.0] *n* = 169	0.005
Haemoglobin, g/dL	13.3 [12.2, 14.3] *n* = 520	13.1 [12.1, 13.9] *n* = 350	14.0 [12.7, 15.1] *n* = 170	<0.001
Number of drugs	5.0 [3.0, 8.0] *n* = 522	5.0 [3.0, 8.0] *n* = 348	5.5 [3.0, 8.0] *n* = 174	0.414
Lumbar T-score	−1.2 [−2.3, 0.1] *n* = 528	−1.8 [−2.6, −0.6] *n* = 354	0.1 [−1.1, 1.5] *n* = 174	<0.001
Femoral-neck T-score	−1.7 [−2.4, −0.9] *n* = 528	−1.9 [−2.5, −1.2] *n* = 354	−1.2 [−2.2, −0.2] *n* = 174	<0.001
ΔT	0.5 [−0.5, 1.4] *n* = 528	0.2 [−0.7, 1.1] *n* = 354	1.1 [0.3, 2.2] *n* = 174	<0.001
Katz ADL	5.0 [4.0, 6.0] *n* = 517	5.0 [4.0, 6.0] *n* = 346	6.0 [4.0, 6.0] *n* = 171	0.010
Lawton IADL	7.0 [3.0, 8.0] *n* = 513	7.0 [3.0, 8.0] *n* = 345	7.0 [3.0, 8.0] *n* = 168	0.092
MMSE	26.0 [22.0, 28.0] *n* = 428	26.0 [22.0, 28.0] *n* = 285	27.0 [24.0, 28.0] *n* = 143	0.169
MNA	12.0 [10.0, 14.0] *n* = 505	12.0 [10.0, 14.0] *n* = 342	12.0 [10.0, 14.0] *n* = 163	0.722
GDS	4.0 [2.0, 7.0] *n* = 435	4.0 [2.0, 8.0] *n* = 291	3.0 [1.0, 5.0] *n* = 144	<0.001
Charlson index	1.0 [1.0, 2.0] *n* = 85	1.0 [1.0, 2.0] *n* = 60	2.0 [0.0, 3.0] *n* = 25	0.728
ACB score	1.0 [0.0, 2.0] *n* = 85	1.0 [0.0, 3.0] *n* = 60	1.0 [0.0, 2.0] *n* = 25	0.964
Diabetes	205/508 (40.4%)	137/342 (40.1%)	68/166 (41.0%)	0.921
Hypertension	363/518 (70.1%)	259/350 (74.0%)	104/168 (61.9%)	0.007
Coronary artery disease	142/499 (28.5%)	76/334 (22.8%)	66/165 (40.0%)	<0.001
Heart failure	50/496 (10.1%)	32/332 (9.6%)	18/164 (11.0%)	0.759
COPD	60/494 (12.1%)	38/330 (11.5%)	22/164 (13.4%)	0.644
Chronic kidney disease	54/495 (10.9%)	31/332 (9.3%)	23/163 (14.1%)	0.148
Dementia	92/496 (18.5%)	59/332 (17.8%)	33/164 (20.1%)	0.609
Depression	93/500 (18.6%)	73/336 (21.7%)	20/164 (12.2%)	0.014
Osteoarthritis	25/490 (5.1%)	19/329 (5.8%)	6/161 (3.7%)	0.454
Stroke	54/495 (10.9%)	32/330 (9.7%)	22/165 (13.3%)	0.284
Hypothyroidism	65/499 (13.0%)	57/335 (17.0%)	8/164 (4.9%)	<0.001
Parkinson disease	25/495 (5.1%)	12/330 (3.6%)	13/165 (7.9%)	0.070
Recorded polypharmacy	307/523 (58.7%)	199/350 (56.9%)	108/173 (62.4%)	0.261
Potentially inappropriate medication	148/488 (30.3%)	95/328 (29.0%)	53/160 (33.1%)	0.404
Statin	111/498 (22.3%)	72/333 (21.6%)	39/165 (23.6%)	0.693
Proton-pump inhibitor	152/488 (31.1%)	104/327 (31.8%)	48/161 (29.8%)	0.732
Falls	192/521 (36.9%)	138/351 (39.3%)	54/170 (31.8%)	0.114
Recorded osteoporosis diagnosis	197/511 (38.6%)	162/345 (47.0%)	35/166 (21.1%)	<0.001
Active OP pharmacotherapy	18/498 (3.6%)	16/334 (4.8%)	2/164 (1.2%)	0.080
OP-threshold discordance	122/528 (23.1%)	96/354 (27.1%)	26/174 (14.9%)	0.003
Femur-worse discordance	65/528 (12.3%)	43/354 (12.1%)	22/174 (12.6%)	0.982
Lumbar-worse discordance	57/528 (10.8%)	53/354 (15.0%)	4/174 (2.3%)	<0.001
Strict Woodson discordance	21/528 (4.0%)	11/354 (3.1%)	10/174 (5.7%)	0.222

Continuous variables are median [IQR], with the available *n* in each cell; binary variables are *n*/*N* (%). Continuous comparisons use two-sided Mann–Whitney U tests; categorical comparisons use Pearson chi-square with Yates correction, or Fisher exact where an expected cell is <5. Missing values were not imputed for descriptive summaries. ΔT was standardised to two decimal places for rank comparisons. Charlson and ACB scores were available for only 85 participants (16.1%); these descriptive summaries are based on a limited subset. Abbreviations: ACB, anticholinergic burden; ADL, activities of daily living; BMI, body mass index; Charlson index, Charlson Comorbidity Index; COPD, chronic obstructive pulmonary disease; eGFR, estimated glomerular filtration rate; GDS, Geriatric Depression Scale; HbA1c, glycated haemoglobin; IADL, instrumental activities of daily living; MMSE, Mini-Mental State Examination; MNA, Mini Nutritional Assessment; OP, osteoporosis; TSH, thyroid-stimulating hormone; TyG, triglyceride–glucose; 25(OH)D, 25-hydroxyvitamin D; ΔT, lumbar minus femoral-neck T-score.

**Table 2:** Hierarchical linear regression of ΔT.

Predictor	B	95% CI	*p*	*p* (HC3)	β	sr^2^	% Total Variance
Age, years	+0.016	−0.001 to +0.033	0.069	0.060	+0.078	0.0057	0.57
Sex (male)	+0.984	+0.730 to +1.238	<0.001	<0.001	+0.319	0.0987	9.87
Diabetes	+0.343	+0.073 to +0.613	0.013	0.015	+0.116	0.0107	1.07
Hypertension	+0.116	−0.144 to +0.377	0.381	0.369	+0.037	0.0013	0.13
TyG index	+0.243	+0.033 to +0.453	0.023	0.032	+0.104	0.0088	0.88

Model 3, *n* = 506. B, unstandardised coefficient; β, standardised coefficient; sr^2^, drop-one semi-partial R^2^. The TyG nested F-test is the primary test. HC3 tests use the asymptotic normal reference. The unique variance contributions do not constitute a partition of model R^2^. Abbreviations: CI, confidence interval; HC3, heteroscedasticity-consistent (type 3) standard errors; TyG, triglyceride–glucose.

**Table 3:** Firth-penalised logistic regression of site discordance.

Outcome/Predictor	OR	95% CI	*p*
Osteoporosis-threshold discordance			
Age, years	1.03	1.00–1.06	0.092
Sex (male)	0.41	0.24–0.67	<0.001
Diabetes	0.67	0.41–1.09	0.109
Hypertension	0.81	0.51–1.30	0.385
TyG index	1.15	0.78–1.68	0.470
Femur-worse discordance			
Age, years	1.04	1.00–1.08	0.036
Sex (male)	0.95	0.52–1.67	0.849
Diabetes	0.93	0.49–1.74	0.828
Hypertension	0.84	0.47–1.53	0.553
TyG index	1.30	0.81–2.07	0.279
Lumbar-worse discordance			
Age, years	1.00	0.96–1.04	0.962
Sex (male)	0.12	0.03–0.31	<0.001
Diabetes	0.51	0.25–1.00	0.051
Hypertension	0.84	0.45–1.59	0.584
TyG index	0.93	0.54–1.58	0.785

All adjusted models use the 506 complete cases (341 women, 165 men). Outcome event totals are 115, 60 and 55, respectively. Lumbar-worse events are 52/341 women and 3/165 men in the adjusted model; the corresponding full-cohort counts are 53/354 and 4/174. Confidence intervals and tests use profile penalised likelihood. Abbreviations: CI, confidence interval; OR, odds ratio; TyG, triglyceride–glucose.

## Data Availability

A de-identified dataset covering the 961 screened records, with analytic-cohort and complete-case flags and an embedded data dictionary, is available from the corresponding author for academic research subject to institutional approval and a data transfer agreement. Participant-level data are not publicly available owing to institutional privacy restrictions. The analysis code (Python), including the Firth implementation and the figure scripts, is openly available under the MIT licence at https://doi.org/10.5281/zenodo.22772661 (version 1.1.3); the code contains no participant data.

## Supplementary Materials

The following supporting information can be downloaded at: https://www.mdpi.com/article/10.3390/jcm15207773/s1, Table S1: Sensitivity analyses for the TyG coefficient; Table S2: Sensitivity analyses for the sex coefficient; Table S3: Included and excluded screened records; Table S4: Exploratory and functional-form analyses; Table S5: Comparison by availability of paired raw BMD; Table S6: Missingness and expanded screened-record comparisons; Table S7: Available field definitions and ascertainment limitations; Figure S1: Sensitivity analyses of the coefficients for (A) male sex and (B) the TyG index; File S1: STROBE and RECORD reporting checklist.
