# Supplementary material for: Correlates of the Lumbar–Femoral T-Score Difference in Older Adults: Sex Outweighs the Triglyceride–Glucose Index
Source: J Clin Med. 2026 Oct 9;15(20):7773. doi: 10.3390/jcm15207773 (PMC13705045; doi:10.3390/jcm15207773)
Supplement: Supplementary file 1 [file jcm-15-07773-s001.zip › jcm-4538017-supplementary File S1.pdf]

Supplementary File S1

STROBE and RECORD reporting checklist

Manuscript jcm-4538017. Correlates of the Lumbar–Femoral T-Score Difference in Older Adults: Sex Outweighs the Triglyceride–Glucose Index.

This checklist maps reporting items to the revised manuscript and supplementary tables. It covers all 22 STROBE items for cross-sectional studies and all 13 RECORD extension items. The entries identify available reporting and unresolved documentation gaps; they do not certify complete compliance or clinical validity. Descriptions are abbreviated; the official checklists are cited below.

Locations use section and table numbers, which remain interpretable across Word and PDF pagination. Supplementary tables refer to the accompanying supplementary document. Where documentation is unavailable, this does not establish that the original procedure was never performed.

STROBE items for cross-sectional studies

Item	Reporting topic	Manuscript location and coverage
1a–b	Design and abstract summary	Abstract. The retrospective cross-sectional design, routine records, study setting, main analyses, findings and limitations are summarised.
2	Rationale	Introduction, paragraphs 1–5. The clinical context, continuous lumbar–femoral T-score difference and rationale for examining sex and TyG are described.
3	Objectives and hypotheses	Introduction, final paragraph; Methods 2.7. TyG remains the prespecified primary exposure and its model contribution the single primary test. The sex emphasis in the presentation does not change this hierarchy; decomposition is identified as post hoc.
4	Design overview	Methods 2.1. The retrospective, single-centre, cross-sectional design is stated early.
5	Setting and dates	Methods 2.1; Abstract. The geriatric outpatient clinic at Ankara Bilkent City Hospital and January–December 2023 are specified. The laboratory-to-DXA interval is unavailable and is acknowledged in Discussion. There was no longitudinal outcome analysis.
6a	Eligibility and selection	Methods 2.2 and 2.6; Results 3.1; Table S6. Selection from 961 screened records to 528 cohort members and 506 primary complete cases is described. Original hospital selection queries and clinical ascertainment algorithms are unavailable (Table S7).
7	Variable definitions	Methods 2.3–2.5; Table S7; workbook dictionary. ΔT, TyG and threshold outcomes are defined. Clinical flags are described as recorded indicators; diagnostic code lists, treatment windows and validation metadata are unavailable.
8	Measurements and comparability	Methods 2.3–2.5; Table S7. Device, reported reference configuration, measured sites and laboratory units are described. A common reference is reported for both sexes. Scan images, laterality, original assay metadata and complete assessment procedures are unavailable.
9	Bias assessment	Methods 2.6–2.7; Results 3.4 and 3.6; Discussion; Tables S1–S7. Quality checks, influence analyses, same-subgroup comparisons, missingness and raw-BMD availability are examined. These analyses do not exclude or bound selection, measurement or residual-confounding bias.

Item	Reporting topic	Manuscript location and coverage
10	Sample-size basis	Methods 2.7. The reported sample-size assumptions, recalculation during revision (minimum 395 participants), and fixed complete-case sample are described. The original G*Power output was unavailable; a publicly archived protocol or dated analysis plan is not identified.
11	Continuous variables and grouping	Methods 2.3, 2.6 and 2.7; Table S4; analysis code. Continuous model terms, ΔT precision for rank tests, log(BMI) imputation, quadratic TyG and exploratory tertiles are specified. Osteoporosis and strict-discordance boundaries are explicit.
12a–e	Models, subgroups, missingness and sensitivity	Methods 2.7; Tables S1–S6. Hierarchical regression, adjustment, robust inference, Firth models, interactions, exploratory analyses and sensitivity specifications are described. Complete-case analysis and BMI-only multiple imputation are distinguished. No complex probability-sampling analysis is reported.
13a–c	Participant flow and exclusions	Results 3.1; Methods 2.2; Table S6. Sequential exclusions and analysis denominators are provided numerically. The screened, analytic and model samples are distinguished. A separate participant-flow figure is not provided; Figures 1–3 show study results.
14a–b	Characteristics and missingness	Table 1; Tables S3, S5–S7; Methods 2.2 and 2.5. Descriptive values are stratified by sex with available denominators. Screened-record missingness, subgroup availability and incomplete eligibility information are disclosed; missing clinical flags are not treated as verified absence.
15	Outcome summaries	Results 3.2 and 3.5; Table 1; Figure 1; Table 3 footnote and Figure 2 caption. Continuous ΔT summaries and categorical event counts are provided. Full-cohort event counts are distinguished from events in the adjusted sample.
16a–c	Estimates, uncertainty and category boundaries	Tables 1–3; Methods 2.3 and 2.7; Results 3.3 and 3.5; Table S4. Unadjusted descriptions and adjusted estimates with confidence intervals are reported, with covariates and outcome thresholds. TyG tertiles are exploratory; their numerical cutpoints are not tabulated. Time-specific absolute-risk conversion is inapplicable because no incident-risk endpoint was studied.
17	Additional analyses	Results 3.4 and 3.6; Tables S1–S6; Figures 3 and S1. Sensitivity analyses, subset comparisons, nonlinear terms, arithmetic decomposition and expanded screened-record comparisons are reported with exploratory or post hoc status where applicable.
18	Findings against objectives	Discussion, opening paragraph; Conclusions. Relative within-model variance contributions are summarised while maintaining the primary TyG analysis hierarchy.
19	Limitations and possible bias	Discussion, limitations paragraphs; Table S7. Selection, unmeasured lumbar degeneration, unavailable laterality, sparse events, residual confounding, timing and incomplete ascertainment documentation are discussed. The direction and magnitude of unresolved selection and measurement bias are not identified by these data.
20	Cautious interpretation	Discussion; Conclusions. Association is separated from causation; exploratory arithmetic components are not causal fractions. Sensitivity, multiple secondary analyses and the absence of fracture or diagnostic-performance endpoints limit interpretation.

Item	Reporting topic	Manuscript location and coverage
21	External validity	Discussion, limitations and raw-BMD subset paragraphs. Generalisation beyond this single-centre clinical cohort, measured subgroup, device and reference implementation is limited.
22	Funding and funder role	Funding statement. No external funding is reported; an external funder role therefore does not arise.

RECORD extension items

Item	Reporting topic	Manuscript location and coverage
1.1	Routine-data source identification	Partly reported. Abstract and Methods 2.1 identify routine geriatric outpatient records. The institution is named in Methods; an original hospital database name or documented database schema is not available.
1.2	Geography and study period	Reported. Abstract identifies Ankara, Türkiye and 2023; Methods 2.1 gives the institution and January–December 2023.
1.3	Linkage disclosed in abstract	Not established. Table S7 states that original cross-database linkage documentation is unavailable. The abstract makes no linkage claim. Absence of that documentation does not establish that no linkage occurred.
6.1	Population-selection algorithms	Partly reported. Methods 2.2 and 2.6, Results 3.1 and Table S6 give reproducible research-extract filters. Table S7 maps cohort flags. Original hospital queries, code lists and eligibility adjudication procedures are unavailable; recorded flags and missing eligibility values retain their stated limitations.
6.2	Selection-algorithm validation	Unavailable. Table S7 and Discussion identify missing source-validation documentation. No validation study of the original selection or clinical-code algorithms is documented. Computational checks of the research extract do not constitute clinical ascertainment validation.
6.3	Linkage flow and counts	Unavailable. Table S7 identifies missing matching identifiers, linked/unlinked counts and linkage-quality records. Table S6 describes research-sample selection; it is not a linkage-flow diagram.
7.1	Codes and derivations for study variables	Partly reported. Methods 2.3–2.5, Table S7 and the workbook dictionary specify available fields, analytic definitions and deterministic outcomes. Original ICD/ATC lists, diagnostic thresholds, look-back windows, source notes and validation records are unavailable. A recorded clinical flag is not represented as a validated ascertainment algorithm.
12.1	Investigators' database access	Partly reported. Methods 2.1 and the ethics statement describe retrospective use of de-identified records. Table S7 identifies unavailable documentation of original institutional database access, abstraction workflow and database-level permissions. The research-extract access does not document full hospital-database access.
12.2	Cleaning and data-quality procedures	Partly reported. Methods 2.6, Table S7, workbook notes, the version-2 and version-3 change logs and validate_data.py document revision corrections, missing-value handling, derivations, structural checks and review triggers. Original scan images and complete source-cleaning records are unavailable. Review flags alone are not evidence that measurements are erroneous.

Item	Reporting topic	Manuscript location and coverage
12.3	Linkage methods and quality	Unavailable. Table S7 states that cross-database linkage methods, identifiers, match counts and quality assessment are undocumented in the retained materials. No deterministic/probabilistic matching method or successful-linkage rate is claimed.
13.1	Derivation of the analytic population	Reported at research-extract level. Methods 2.2 and 2.6, Results 3.1 and Table S6 describe exclusions for age, measurement availability, recorded clinical exclusions and DXA plausibility, followed by model complete-case selection. Original database extraction and linkage restrictions cannot be reconstructed.
19.1	Consequences of routine-data limitations	Reported with unresolved gaps. Discussion and Table S7 address unavailable ascertainment metadata, possible misclassification, missing measurements, unmeasured confounding, selection, lumbar degeneration, laterality and laboratory/DXA timing. Changes in original coding or eligibility over 2023 cannot be evaluated from the retained extract.
22.1	Access to protocol, data and code	Partly reported. The Data Availability Statement describes controlled access to participant-level data and gives the repository identifier of the openly available Python code and notebook (https://doi.org/10.5281/zenodo.22772661). The workbook contains a dictionary. A public protocol identifier is not documented.

Checklist sources

STROBE Initiative. Cross-sectional checklist. https://www.strobe-statement.org/download/strobe-checklist-cross-sectional-studies-pdf

Benchimol EI, Smeeth L, Guttmann A, et al. The RECORD Statement. PLoS Medicine. 2015;12:e1001885. https://doi.org/10.1371/journal.pmed.1001885
