# Supplementary material for: Correlates of the Lumbar–Femoral T-Score Difference in Older Adults: Sex Outweighs the Triglyceride–Glucose Index
Source: J Clin Med. 2026 Oct 9;15(20):7773. doi: 10.3390/jcm15207773 (PMC13705045; doi:10.3390/jcm15207773)
Supplement: Supplementary file 1 [file jcm-15-07773-s001.zip › jcm-4538017-supplementary tables and figure.pdf]

Supplementary Materials

Correlates of the Lumbar–Femoral T-Score Difference in Older Adults: Sex Outweighs the Triglyceride–Glucose Index

Rıdvan Erten and co-authors

Tables S1–S7 and Figure S1 accompany manuscript jcm-4538017. The analyses distinguish the 528-person descriptive cohort, the fixed 506-person primary regression sample and smaller subsets with additional measurements. The separate Supplementary File S1 contains the STROBE and RECORD checklist.

Table S1. Sensitivity analyses for the TyG coefficient

Specification	n	B (TyG)	95% CI	p
Primary model	506	+0.243	+0.033 to +0.453	0.023
Primary model with HC3 inference	506	+0.243	+0.022 to +0.465	0.032
Diabetes omitted, same sample	506	+0.348	+0.154 to +0.542	<0.001
BMI imputation during revision	506	+0.240	+0.030 to +0.450	0.025
No documented active OP treatment	490	+0.276	+0.064 to +0.488	0.011
BMI adjusted	348	+0.209	−0.027 to +0.446	0.082
Influential observations excluded	473	+0.070	−0.115 to +0.255	0.458
Two flagged DXA records retained	508	+0.214	−0.010 to +0.438	0.061
Raw-BMD availability adjusted	506	+0.250	+0.038 to +0.461	0.021
Lumbar minus femoral neck, hip subset	106	−0.070	−0.531 to +0.390	0.763
Lumbar minus total hip, same subset	106	−0.037	−0.515 to +0.441	0.878
Smoking and alcohol adjusted	103	−0.011	−0.512 to +0.489	0.964
Clinical frailty adjusted	108	−0.056	−0.525 to +0.414	0.815
Height adjusted	358	+0.178	−0.055 to +0.410	0.134
Height and weight adjusted	349	+0.182	−0.051 to +0.414	0.125
Recorded osteoarthritis adjusted	490	+0.309	+0.091 to +0.527	0.006

All models adjust for age, sex, diabetes and hypertension unless a variable is explicitly omitted. BMI, height, smoking/alcohol, frailty and osteoarthritis models additionally include the named variables. HC3 inference uses the asymptotic normal reference. The revision BMI-imputation model uses 50 posterior imputations of log(BMI), exponentiated before BMI-adjusted OLS; pooling uses Rubin's rules with Barnard–Rubin degrees of freedom. This specification is newly documented during revision and does not address missing DXA. The corrected workbook and accompanying Python code were used for all results reported here.

Table S2. Sensitivity analyses for the sex coefficient

Specification	n	B (male)	95% CI	p
Primary model	506	+0.984	+0.730 to +1.238	<0.001

Specification	n	B (male)	95% CI	p
Primary model with HC3 inference	506	+0.984	+0.725 to +1.244	<0.001
Diabetes omitted, same sample	506	+0.994	+0.738 to +1.249	<0.001
BMI imputation during revision	506	+1.010	+0.752 to +1.267	<0.001
No documented active OP treatment	490	+1.023	+0.767 to +1.279	<0.001
BMI adjusted	348	+1.100	+0.806 to +1.394	<0.001
Influential observations excluded	473	+0.873	+0.653 to +1.093	<0.001
Two flagged DXA records retained	508	+0.999	+0.726 to +1.272	<0.001
Raw-BMD availability adjusted	506	+0.984	+0.730 to +1.239	<0.001
Lumbar minus femoral neck, hip subset	106	+0.647	+0.043 to +1.251	0.036
Lumbar minus total hip, same subset	106	+0.331	−0.295 to +0.958	0.297
Smoking and alcohol adjusted	103	+0.228	−0.411 to +0.866	0.481
Clinical frailty adjusted	108	+0.574	−0.026 to +1.175	0.061
Height adjusted	358	+0.711	+0.333 to +1.090	<0.001
Height and weight adjusted	349	+0.669	+0.291 to +1.047	<0.001
Recorded osteoarthritis adjusted	490	+0.960	+0.702 to +1.217	<0.001
Paired coefficient difference (FN outcome minus TH outcome)	106	+0.315	+0.040 to +0.591	0.025

The sex coefficient compares men with women. Wide intervals in smaller subgroups should not be interpreted as evidence of no association. Full-cohort and restricted-sample coefficients need not target identical populations. The paired coefficient difference is estimated within the same 106 participants by regressing total-hip minus femoral-neck T-score on the primary covariates; its 95% confidence interval (+0.040 to +0.591) excludes zero; the selected subgroup limits generalisability.

Table S2, continued. Same-subgroup comparisons before and after adding covariates

Added covariate(s)	Same n	Sex B before → after	TyG B before → after
BMI	348	+1.061 → +1.100	+0.214 → +0.209
Height	358	+1.086 → +0.711	+0.197 → +0.178
Smoking and alcohol	103	+0.439 → +0.228	+0.006 → −0.011
Frailty	108	+0.597 → +0.574	−0.051 → −0.056
Osteoarthritis	490	+0.961 → +0.960	+0.312 → +0.309

Table S3. Included and excluded screened records

Characteristic	Included (n = 528)	Excluded (n = 433)	p
Age, years	76.0 [72.0, 82.0]<br>n = 528	77.0 [72.0, 83.0]<br>n = 432	0.545
Sex (male)	174/528 (33.0%)	178/433 (41.1%)	0.011

Age is available for 432 excluded records; one excluded record lacks age. The 433 excluded records include all screening exclusions, of which 365 were excluded for missing core measurements after the initial age screen. A sex imbalance in availability raises the possibility of selection bias; age similarity does not establish its absence.

Table S4. Exploratory and functional-form analyses

Analysis / term	n	B	95% CI	p
Linear TyG, Model 3	506	+0.243	+0.033 to +0.453	0.023
Quadratic TyG term	506	−0.015	−0.216 to +0.185	0.881
TyG tertile indicator (TyG_tertile2)	506	+0.075	−0.221 to +0.371	0.619
TyG tertile indicator (TyG_tertile3)	506	+0.284	−0.026 to +0.595	0.073
Reference-free log BMD ratio, male	108	+0.017	−0.056 to +0.091	0.637
Age, lumbar T-score	506	−0.025	−0.045 to −0.004	0.018
Age, femoral-neck T-score	506	−0.041	−0.054 to −0.028	<0.001

Quadratic models add squared mean-centred TyG; tertile indicators replace continuous TyG. Reference-free log-ratio and site-specific age models adjust for age, sex, diabetes, hypertension and TyG as applicable. The log-ratio analysis includes 108 patients with paired raw BMD and complete model covariates. Its wide confidence interval is not evidence of no association.

Table S5. Comparison by availability of paired raw BMD

Characteristic	Raw BMD (n = 130)	No raw BMD (n = 398)	p
Age, years	75.0 [71.2, 80.0]<br>n = 130	77.0 [72.0, 83.0]<br>n = 398	0.017
Sex (male)	41/130 (31.5%)	133/398 (33.4%)	0.773
BMI, kg/m²	29.6 [25.0, 34.2]<br>n = 92	27.6 [24.0, 31.2]<br>n = 269	0.006
Diabetes	55/110 (50.0%)	150/398 (37.7%)	0.026
Hypertension	98/120 (81.7%)	265/398 (66.6%)	0.002
TyG index	8.8 [8.3, 9.1]<br>n = 130	8.9 [8.5, 9.3]<br>n = 398	0.015
Lumbar T-score	-0.7 [-1.9, 0.5]<br>n = 130	-1.4 [-2.4, -0.1]<br>n = 398	0.003
Femoral-neck T-score	-1.3 [-2.2, -0.6]<br>n = 130	-1.8 [-2.5, -1.1]<br>n = 398	<0.001
ΔT	0.6 [-0.5, 1.4]<br>n = 130	0.5 [-0.5, 1.4]<br>n = 398	0.779

Continuous data are median [IQR] with available n; binary data are n/N (%). Tests use Mann–Whitney U for continuous variables and chi-square/Fisher tests for binary variables. ΔT is recalculated from the site T-scores and standardised to two decimal places before ranking. This preserves recorded two-decimal T-scores and resolves floating-point differences in ties; p = 0.779091, reported as 0.779. Non-significant ΔT and sex comparisons do not establish equivalence or representativeness.

Table S6. Missingness and expanded screened-record comparisons

A. Core-variable missingness among 961 screened records

Variable	Missing, n (%)	Women missing	Men missing	Age p	Sex p
Lumbar T-score	299 (31.1%)	163/609 (26.8%)	136/352 (38.6%)	0.887	<0.001
Femoral-neck T-score	301 (31.3%)	166/609 (27.3%)	135/352 (38.4%)	0.756	<0.001

Variable	Missing, n (%)	Women missing	Men missing	Age p	Sex p
Glucose, mg/dL	40 (4.2%)	25/609 (4.1%)	15/352 (4.3%)	0.870	1.000
Triglyceride, mg/dL	107 (11.1%)	70/609 (11.5%)	37/352 (10.5%)	0.883	0.719
Diabetes	47 (4.9%)	28/609 (4.6%)	19/352 (5.4%)	0.543	0.690
Hypertension	26 (2.7%)	15/609 (2.5%)	11/352 (3.1%)	0.634	0.687

Age p compares available age in records with versus without the field using Mann–Whitney U. Sex p compares field-missingness frequencies. Age was available in 960 of 961 records. These comparisons describe observed associations and do not identify the missing-data mechanism.

B. Joint availability and reasons for exclusion

Paired site T-scores	Glucose and triglyceride	All screened n
Available	Both available	592
Available	One or both missing	60
Unavailable	Both available	255
Unavailable	One or both missing	54

Among the 309 records without paired T-scores, both fields were missing in 291, only lumbar T-score in 8 and only femoral-neck T-score in 10. Among the 124 excluded records with paired T-scores, 60 lacked glucose or triglyceride, 51 had recorded active malignancy, 24 had eGFR <30, 2 were aged <65, 1 lacked age and 2 met the DXA plausibility-exclusion rule. These reasons overlap and are not mutually exclusive. After the initial age screen, 589 records had paired scores and both laboratory measurements. Missing measurement fields do not establish that a DXA scan was never performed.

C. ΔT among included and excluded records with paired T-scores

Sex	Included	Excluded with paired scores	p
Women	0.2 [-0.7, 1.1]<br>n = 354	0.6 [-0.4, 1.4]<br>n = 83	0.037
Men	1.1 [0.3, 2.2]<br>n = 174	1.0 [0.2, 2.3]<br>n = 41	0.907

Two-sided Mann–Whitney tests use standardised ΔT. The comparison includes excluded records that fail other clinical or quality criteria and is descriptive.

D. Age-adjusted sex estimates in expanded paired-score samples

Sample	n	B (male)	95% CI	p
Analytic cohort	528	+1.014	+0.764 to +1.264	<0.001
All screened records with paired scores	651	+0.976	+0.737 to +1.216	<0.001
Paired scores retained by recorded clinical and quality filters	574	+0.968	+0.724 to +1.211	<0.001

The all-paired model uses 651 of 652 records because one lacks age. The comparison retaining the recorded clinical and quality filters adds 46 records to the 528-person cohort (n = 574), excluding age <65, recorded eGFR <30, recorded active malignancy and flagged DXA values. Missing eGFR or active-malignancy status does not itself trigger exclusion; 5 and 38 analytic-cohort records, respectively, lack these fields. Their eligibility cannot be fully confirmed from the extract. In the age-adjusted all-paired model with sex, inclusion and their interaction, the sex-by-inclusion coefficient is +0.130 (95% CI −0.478 to +0.738; p = 0.674). Neither a small change in a pooled coefficient nor a non-significant interaction estimates or bounds selection bias. The direction and magnitude of bias among records without paired T-scores remain unknown.

Table S7. Available field definitions and ascertainment limitations

The column names below belong to the supplied deidentified research extract, not to a documented hospital database schema. The extract contains derived clinical flags but does not contain the underlying diagnostic code lists, original hospital field names, source-note text, ascertainment windows, or validation records. Binary flags are recorded indicators; a zero is not independent confirmation of clinical absence. Missing values remain missing. The clinical labels do not establish the original ICD or ATC coding. Documentation of institutional database access, abstraction workflow and cross-database linkage, including matching identifiers, match counts and linkage-quality assessment, is unavailable in the supplied materials. Selection and ascertainment validation are likewise not documented. These gaps do not establish that the original procedures or validation never existed.

Variable	Research-extract field	Definition and available ascertainment information
Research identifier	study_id	Permuted identifier 1–961; no institutional meaning according to the dictionary. Not an institutional record number or a documented linkage key.
Eligibility before DXA quality exclusions	in_analysis_set	Boolean flag identifying 530 records in the supplied dictionary. Research selection flag; hospital selection query and original eligibility adjudication are not supplied.
DXA quality exclusion	qc_impossible_dxa	Boolean flag for the two records excluded by the manuscript's DXA plausibility limits. Threshold-based data-quality flag; source images are unavailable for independent adjudication.
Analytic cohort	in_analytic_cohort	Boolean flag identifying 528 records after the documented selection process. Age, core-measurement availability and recorded eGFR, active-malignancy and DXA-quality filters are described in Methods. Missing eGFR (5 cohort records) or active-malignancy status (38) did not trigger exclusion; clinical eligibility cannot be fully confirmed for these records.
Primary complete cases	complete_case_primary	Boolean flag identifying 506 cohort records complete for ΔT, age, sex, DM, HT and TyG. The adjusted model denominator differs from the full cohort denominator.
Age	age	Recorded age in years; the analytic eligibility threshold is ≥65 years. Date used for age calculation and original birth-date field are not supplied.
Sex	sex_male	Binary analytic indicator: 1 male, 0 female. Original hospital field name and recording procedure are not supplied.
TyG components and index	glucose; triglyceride; TyG_calc	Glucose and triglycerides in mg/dL; TyG = ln[(triglyceride × glucose)/2]. The manuscript describes fasting samples; fasting verification, assay metadata and laboratory–DXA intervals are not available in the extract.
Lumbar and femoral-neck T-scores	lumbar_T; femoral_neck_T	Device-reported T-scores; lumbar score is the L1–L4 composite. Device/reference details appear in Methods 2.3. Vertebra-level images and femoral scan laterality are unavailable. Two source values have two decimal places and are preserved.
Primary continuous outcome	dT_calc; lumbar_T; femoral_neck_T	ΔT is recomputed as lumbar_T − femoral_neck_T; numeric precision is standardised to two decimal places for rank tests. Stored dT_calc can contain floating-point differences from recomputation. Precision standardisation is not source-data correction.
Total-hip sensitivity outcome	total_hip_T; lumbar_T	Lumbar–total-hip difference = lumbar_T − total_hip_T. Total-hip T-score is available for 127 cohort members; 106 have complete primary model covariates.

Variable	Research-extract field	Definition and available ascertainment information
Raw areal BMD	lumbar_BMD; femoral_neck_BMD	Areal BMD in g/cm²; both sites are available in 130 cohort members. The data-version-2 and data-version-3 change logs document source-reviewed corrections and unverifiable values set to missing; data version 3 reinstates one verified femoral-neck BMD value. Original scan images are not included in the deidentified extract.
Any osteoporosis-threshold discordance	any_discordance; lumbar_T; femoral_neck_T	Indicator that exactly one site has T-score ≤−2.5; re-derived from the two T-scores. Not equivalent to all classic minor/major discordance categories; missing if either site is missing.
Femur-worse discordance	femur_worse; lumbar_T; femoral_neck_T	1 if femoral_neck_T ≤−2.5 and lumbar_T >−2.5; otherwise 0 when both sites are known. Derived threshold outcome, not a diagnostic-code flag.
Lumbar-worse discordance	lumbar_worse; lumbar_T; femoral_neck_T	1 if lumbar_T ≤−2.5 and femoral_neck_T >−2.5; otherwise 0 when both sites are known. Derived threshold outcome; full-cohort and adjusted-model event counts differ.
Strict discordance	woodson_strict; lumbar_T; femoral_neck_T	1 if one site has T-score ≥−1.0 and the other ≤−2.5; otherwise 0 when both sites are known. Recomputed threshold indicator; not based on ICD codes.
Diabetes	DM	Recorded binary clinical indicator, 0/1; missing retained. Diagnostic thresholds, ICD lists, medication criteria, ascertainment window and source validation were not supplied.
Hypertension	HT	Recorded binary clinical indicator, 0/1; missing retained. Blood-pressure criteria, treatment rules, ICD lists, ascertainment window and source validation were not supplied.
Renal function and recorded CKD	eGFR; CKD	eGFR in mL/min/1.73 m², described as CKD-EPI in the manuscript; recorded values <30 trigger exclusion. Five analytic-cohort records lack eGFR and were retained. CKD is a separate recorded 0/1 flag. Creatinine inputs, equation implementation and CKD chronicity/diagnostic algorithm are not supplied; eGFR and CKD are not interchangeable.
Active malignancy	cancer_active	Recorded 0/1 indicator; value 1 contributes to exclusion. Thirty-eight analytic-cohort records lack this field and were retained; missing status is not confirmation of absence. Definition of active disease, relevant dates, tumour codes and source validation were not supplied.
Osteoarthritis	osteoarthritis	Recorded 0/1 indicator used in the additional sensitivity analysis. Anatomic site and severity are not encoded; the flag is not a vertebra-level measure of lumbar degeneration.
Cardiovascular and cerebrovascular diagnoses	CAD; CHF; stroke	Separate recorded 0/1 indicators for coronary artery disease, heart failure and stroke. Original diagnosis codes, diagnostic criteria, subtype, timing and validation are not supplied.
Other clinical diagnoses	COPD; dementia; parkinson; hypothyroid; depression_dx	Separate recorded 0/1 indicators; missing values remain missing. Original code lists, criteria, ascertainment windows and validation are not supplied.
Recorded osteoporosis diagnosis	OP_diagnosis	Recorded 0/1 diagnosis flag, separate from recomputed T-score thresholds. Coding algorithm and relation to historical fractures, previous DXA or treatment are not supplied.

Variable	Research-extract field	Definition and available ascertainment information
Body size	height_clean; weight_kg; BMI_clean	Height in cm, weight in kg and BMI in kg/m². Data version 2 includes a body-weight correction documented as source verified in the change log. Earlier height/BMI cleaning is not fully documented in the minimal extract; recorded versus computed BMI cannot be distinguished for every record.
Laboratory descriptors	hba1c; calcium; albumin; vitamin_D; TSH; hemoglobin	Recorded laboratory measures; vitamin_D represents 25(OH)D in nmol/L, albumin is harmonised to g/L, and HbA1c is reported as %. The data-version-2 change log documents albumin unit harmonisation. Two HbA1c values initially set to missing were subsequently confirmed as IFCC mmol/mol and converted to NGSP % in data version 3. Original assay/source-unit records and sampling dates are not included. Vitamin D is descriptive and is not a primary model covariate.
Medication count and polypharmacy	n_drugs; polypharmacy	Recorded medication count and separate 0/1 polypharmacy flag; the stored flag is retained for descriptive summaries. Among 521 analytic-cohort records with both fields observed, 14 stored flags disagree with a threshold of ≥5 recorded drugs. Source timing and classification rules are unavailable, so these discrepancies cannot be reconciled from the extract.
Potentially inappropriate medication	PIM	Recorded 0/1 indicator. Assessment instrument, version and medication criteria are not supplied.
Statin and proton-pump inhibitor use	statin; PPI	Separate recorded 0/1 medication indicators. Drug/ATC lists, prescribed-versus-active use, dose, duration and ascertainment window are not supplied.
Osteoporosis pharmacotherapy	OP_pharmacotherapy	Recorded 0/1 treatment indicator; missing status remains unknown. Drug names, ATC codes and treatment-window criteria are unavailable. The extract does not establish whether undocumented treatment status represents verified non-use.
Frailty	CFS	Recorded Clinical Frailty Scale score; intended integer range 1–9. Three zero-coded entries (two in the analytic cohort) were set to missing in data version 2 because their source scores could not be verified. The adjusted frailty model includes 108 complete cases. Rater, version, assessment date and original scoring form are not supplied.
Smoking and alcohol	smoking; alcohol	Separate recorded 0/1 indicators used in sensitivity analysis. Current-versus-ever definitions, quantity, duration and assessment dates are not supplied.
Geriatric and comorbidity scores	Katz; Lawton; MMSE; MNA; GDS; CCI; ACB_score	Recorded score variables retained for descriptive or supplementary comparisons. Instrument versions, scoring algorithms, assessors and source forms are not supplied; names alone do not establish score validity.
Falls	falls	Recorded 0/1 indicator. Recall period, number of falls and ascertainment procedure are not supplied.

Sensitivity analyses

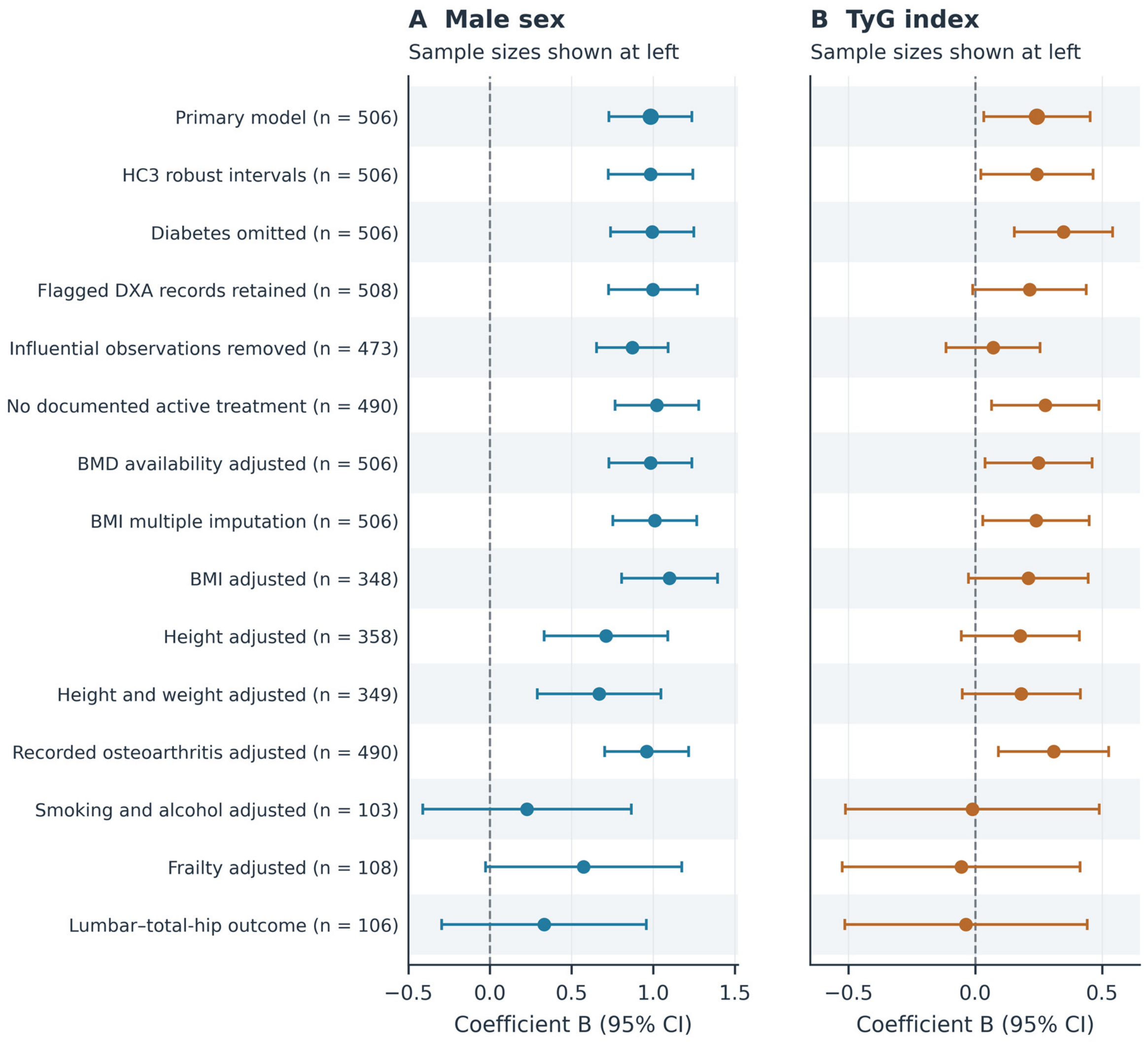

Figure S1. Sensitivity analyses of the coefficients for (A) male sex and (B) the TyG index. Points and horizontal bars show unstandardised coefficients and 95% confidence intervals; the dashed line denotes zero. Each panel uses the sample sizes shown beside the model specifications. Except for the HC3 and multiple-imputation rows, intervals are from ordinary least squares; the primary adjustment set includes age, sex, diabetes, hypertension and TyG. Subgroup estimates use different participant sets, and changes from the full-sample estimate may reflect both sample composition and model specification. Corresponding models within the same subsets are reported in Tables S1 and S2. The last row uses lumbar minus total-hip T-score as the outcome.
